# A mercury complex-based fluorescent sensor for biological thiols†

**DOI:** 10.1039/d5ra02268a

**Published:** 2025-06-13

**Authors:** Nguyen Khoa Hien, Trinh Thi Giao Chau, Nguyen Dinh Luyen, Quan V. Vo, Mai Van Bay, Son Tung Ngo, Pham Cam Nam, Duong Tuan Quang

**Affiliations:** a Mientrung Institute for Scientific Research, Vietnam National Museum of Nature, Vietnam Academy of Science and Technology Hue 49000 Vietnam; b Department of Chemistry, Hue University Hue 49000 Vietnam duongtuanquang@dhsphue.edu.vn; c Faculty of Chemical Technology-Environment, The University of Danang - University of Technology and Education 48 Cao Thang Danang 50000 Vietnam; d The University of Danang, University of Science and Education Danang 50000 Vietnam; e Laboratory of Biophysics, Institute for Advanced Study in Technology, Ton Duc Thang University Ho Chi Minh City 72915 Vietnam; f Faculty of Pharmacy, Ton Duc Thang University Ho Chi Minh City 72915 Vietnam; g The University of Danang, University of Science and Technology Danang 50000 Vietnam pcnam@dut.udn.vn

## Abstract

A novel fluorescent sensor, Hg(DST)_2_, was developed for the selective detection of biological thiols, including glutathione (GSH), cysteine (Cys), and homocysteine (Hcy), in fully aqueous solutions at pH 7.2. The sensor exhibited significant fluorescence quenching upon coordination with Hg^2+^, which was reversibly restored in the presence of thiols due to the formation of thermodynamically favored Hg-thiol complexes. The OFF–ON fluorescence mechanism of the sensor was elucidated using DFT calculations. Fluorescence titration experiments revealed a strong linear correlation (*R*^2^ ≈ 0.998) between fluorescence intensity and thiol concentrations within the ranges of 0.34–8.00 μM for GSH, 0.47–10.00 μM for Cys, and 0.26–8.00 μM for Hcy, with corresponding limits of detection (LOD) of 0.34, 0.47, and 0.26 μM, respectively. The sensor demonstrated high selectivity toward thiols in the presence of common amino acids, metal ions, and anions, with interference from Ag^+^, Cu^2+^, Co^2+^, and Ni^2+^ mitigated using 1,10-phenanthroline (PHEN). Owing to its high sensitivity, selectivity, and water solubility, Hg(DST)_2_ represents a promising tool for thiol quantification in biological and environmental matrices.

## Abstract 2

A novel fluorescent sensor, Hg(DST)_2_, was developed for the selective detection of biological thiols, including glutathione (GSH), cysteine (Cys), and homocysteine (Hcy), in fully aqueous solutions at pH 7.2.

## Graphical abstract

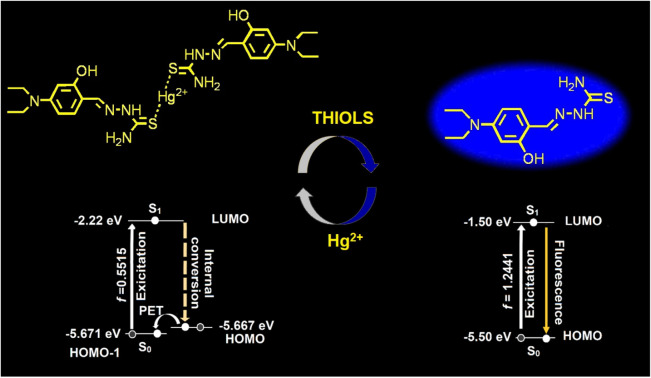

## 1. Introduction

Cysteine (Cys), glutathione (GSH), and homocysteine (Hcy) are non-protein biological thiols that play crucial roles in various physiological and biochemical functions in humans.^1–3^ In particular, Cys is a precursor for protein synthesis and plays a vital role in protecting cells from damage caused by free radicals and oxidative agents. Additionally, Cys is involved in detoxification processes by binding to heavy metals and other toxic substances, aiding in their removal from the body.^4^ The intracellular concentration of Cys ranges from 30 to 200 μM.^5^ The highest concentration of Cys in plasma has been found to reach up to 250 μM.^1^ Changes in Cys levels have been shown to be associated with several diseases such as Alzheimer's and Parkinson's disease, autoimmune deficiency syndrome, and hyperhomocysteinemia.^1^

Glutathione is a tripeptide made up of three amino acids: l-glutamate, l-cysteine, and l-glycine. It is the most abundant non-protein thiol in cells, with intracellular concentrations ranging from 1 to 15 mM, primarily in its reduced form.^6^ GSH is one of the body's most powerful antioxidants, protecting cells from oxidative damage by neutralizing free radicals.^6,7^ It plays a critical role in detoxification, particularly in the liver, where it helps eliminate harmful substances and convert them into less toxic forms.^8^ Additionally, GSH is essential for maintaining and regulating the immune system.^6^ It is also believed to be associated with diseases, including cancer, stroke, heart disease, pancreatic and kidney disorders, diabetes, Alzheimer's, Parkinson's, gastritis, peptic ulcers, and atherosclerosis diseases.^6,7^

Homocysteine (Hcy) is a metabolic intermediate formed during the catabolism of methionine in the one-carbon metabolism cycle. It participates in methylation processes, methionine regeneration, and cysteine biosynthesis, with its homeostasis maintained through pathways that either regenerate methionine or convert it to cysteine.^9^ Typical blood Hcy levels range from 5 to 13 μM.^10^ Elevated Hcy levels, a condition known as hyperhomocysteinemia, are linked to cardiovascular, neurological, and bone-related disorders, as well as fertility issues, including recurrent miscarriages and infertility in both genders.^11–14^

Given the important roles of Cys, GSH, and Hcy, various methods for their determination have been developed, including high-performance liquid chromatography,^1,15^ gas chromatography-mass spectrometry,^16^ electrochemistry,^17^ and UV-Vis absorption and fluorescence spectroscopy.^18–20^ Among these, fluorescent sensors have garnered significant interest from scientists due to their high sensitivity, simple analysis methods, and ability to monitor in living cells.^21–24^

Some recently reported fluorescent sensors for biothiol detection based on metal ion complexes are summarized in Table 1. Most reported fluorescent sensors do not selectively detect individual biothiols because Cys, GSH, and Hcy have similar structures.^25^ Nevertheless, this limitation does not diminish the efforts to develop new fluorescent sensors for biothiols. This is because, in practice, there are cases where only the concentration change of a specific biothiol related to a particular disease is of interest, such as monitoring glutathione levels in the liver to assess liver function or monitoring homocysteine levels in the blood to evaluate the risk of cardiovascular diseases and stroke.^26–28^ Additionally, different biothiols are distributed differently in various body parts. For example, the concentrations of cysteine and glutathione are highest in tissues, particularly in the liver, while homocysteine is usually undetectable in tissues and is primarily found in plasma.^29–31^ Notably, fluorescent sensors often interact reversibly with biothiols, which is very useful for monitoring real-time changes in biothiol concentrations, and providing detailed information about pathological conditions.^10^

**Table 1:** Some fluorescent sensors for biothiol detection based on metal ion complexes

Complexes of metal ions	Detectable biothiols	LOD (μM)	Solvent/pH	The influence of other thiols	Ref.
Cu^2+^	GSH	0.16	HEPES/DMSO (95/5, v/v), pH 7.4	Affected by Cys and Hcy	32
Cu^2+^	GSH	0.80	DMF/HEPES (3/7, v/v), pH 7.4	Cannot detect individual thiols separately	33
	Cys	1.00			
	Hcy	1.50			
Cu^2+^	GSH	0.30	Ethanol/HEPES (1/1, v/v), pH 7.4	Cannot detect individual thiols separately	19
	Cys				
	Hcy				
Cu^2+^	Cys	0.17	CH_3_CN/HEPES (1/1, v/v), pH 7.4	- Cannot detect individual thiols separately	34
	Hcy	0.25		- The influence of GSH has not been reported	
Cu^2+^	GSH	0.20	CH_3_OH/HEPES (9/1, v/v), pH 7.2	The influence of Cys and Hcy has not been reported	35
Cu^2+^	GSH	0.44	DMF/HEPES (7 : 3, v/v), pH 7.4	Cannot detect individual thiols separately	36
	Cys	0.96			
	Hcy	0.68			
Cu^2+^	Cys	0.015	CH_3_CN/HEPES (7/3, v/v), pH 7.4	The influence of GSH and Hcy has not been reported	37
Hg^2+^	Cys	0.20	Ethanol/HEPES (1 : 9, v/v), pH 7.4	Affected by GSH and Hcy	20
Hg^2+^	Cys	0.016	CH_3_CN	The influence of GSH and Hcy has not been reported	38
Ag^+^	GSH	0.208	Dioxane/Tris–HClO_4_ (3/7, v/v), pH 7.4	Cannot detect individual thiols separately	39
	Cys	0.089			
	Hcy	0.174			
Fe^3+^	Cys	0.45	Water (1% DMSO) pH: 2–11	Not affected by GSH and Hcy	40

In this study, we report a fluorescent sensor (Hg(DST)_2_) that can detect the real-time concentrations of Cys, GSH, and Hcy, based on the complex of a fluorescent compound DST with Hg^2+^ ions. The Hg(DST)_2_ complex interacts reversibly with Cys, GSH, and Hcy, releasing DST, accompanied by a fluorescence signal change from *OFF* to *ON* at 445 nm. This process can be reversed at least five times by alternating the addition of Hg^2+^ and biothiols.

## 2. Materials and methods

### 2.1. Instruments

The structural characteristics of the compounds were determined through the study of ^1^H NMR, ^13^C NMR, and mass spectra, using a Bruker Ascend 600 spectrometer for NMR and an Agilent 6200 series TOF LC/MS system for mass spectrometry. The absorption and fluorescence properties of the compounds were studied using Shimadzu spectrometers, with the UV-1800 for UV-Vis absorption spectra and the RF-5301 PC Series for fluorescence spectra.

### 2.2. Reagents

All chemicals used were purchased from Merck. Among them, 4-(diethylamino)salicylaldehyde and aminothiourea were of synthesis grade. The solvents used were of HPLC grade. The metal salts, amino acids, and biothiols (Cys, GSH, and Hcy) were of analytical purity grade.

### 2.3. Computational methodology

Density Functional Theory (DFT) calculations were performed using the Gaussian 16 software at the PBE0/Lanl2dz level of theory.^41–45^ Time-Dependent Density Functional Theory (TD-DFT) method was employed to investigate the excited states.^45,46^ Solvent calculations were carried out using the Polarizable Continuum Model (PCM).^47^

## 3. Results and Discussion

### 3.1. Synthesis and characterization of the Hg(DST)_2_ sensor

**Fig. 1:**
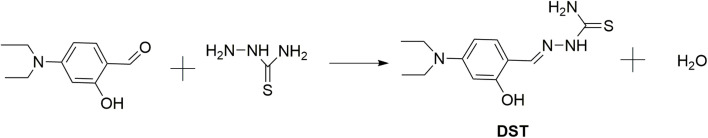
The reaction scheme for the synthesis of the DST fluorescent compound.

The DST fluorescent compound was synthesized according to the reaction shown in Fig. 1, as previously reported.^48^ Briefly, equimolar solutions of aminothiourea and 4-(diethylamino)salicylaldehyde were refluxed in ethanol with anhydrous sodium sulfate at 85 °C under a nitrogen atmosphere for 8 hours, followed by stirring at ambient temperature for 12 hours. The reaction mixture was filtered to remove sodium sulfate, purified *via* silica gel column chromatography using a chloroform : methanol (70 : 1, v/v) eluent, and the solvent was evaporated under reduced pressure, yielding DST with an approximate efficiency of 71.4% (^13^C NMR, ^1^H NMR, and MS spectra are provided in Fig. S1–S3 of the ESI data†).

**Fig. 2:**
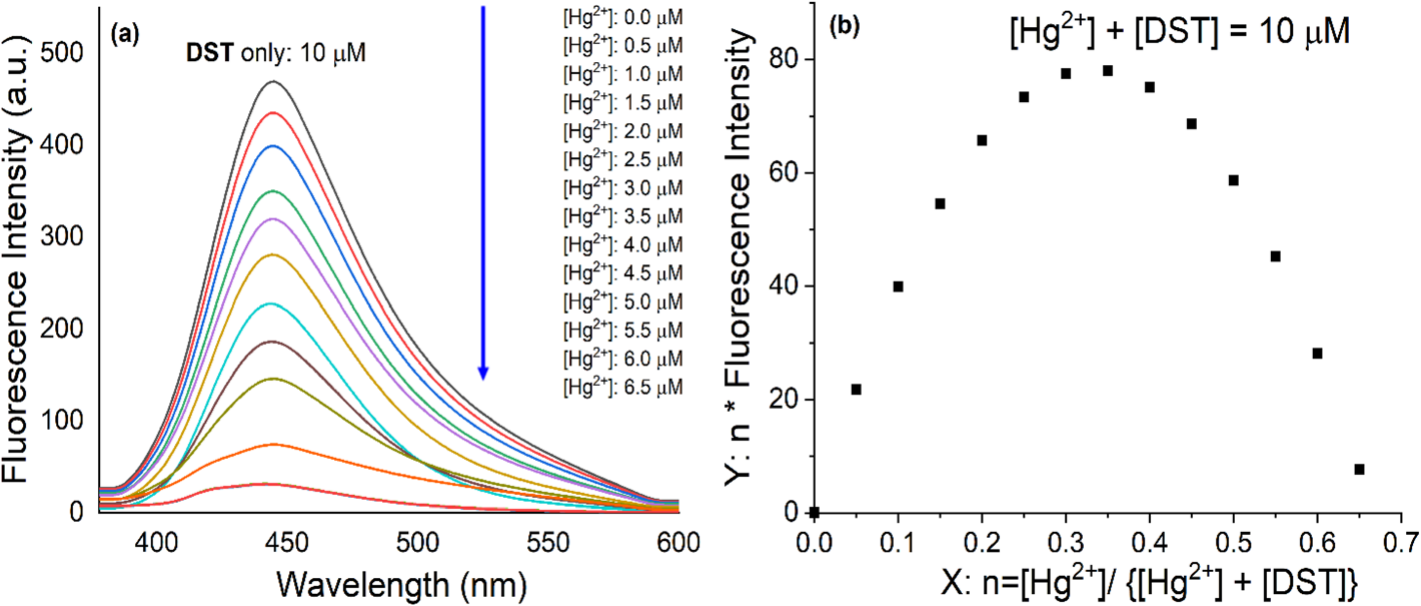
(a) The fluorescence titration spectra of DST solution (10 μM) with Hg^2+^ at concentrations ranging from 0 to 6.5 μM; (b) Job's plot of the interaction between Hg^2+^ and DST (in pH 7.2 phosphate buffer, with an excitation wavelength of 368 nm, an emission wavelength of 445 nm).

Experimental results in Fig. 2 showed that DST exhibited a fluorescence spectrum with a maximum wavelength at 445 nm, with an excitation wavelength at 368 nm. DST reacted with Hg^2+^ in a 2 : 1 molar ratio, accompanied by approximately 95% fluorescence quenching. The Stern–Volmer plot for the fluorescence quenching of DST by Hg^2+^ (Fig. S4†) showed that the relationship between the ratio *F*_0_/*F* and the concentration *Q* of Hg^2+^ (where *F*_0_ and *F* were the fluorescence intensities of the DST solution in the absence and presence of Hg^2+^ at concentration *Q*, respectively) was not linear but exhibited an upward (positive) deviation. This suggested that both static and dynamic quenching mechanisms were responsible for the observed decrease in fluorescence intensity.^49,50^

**Fig. 3:**
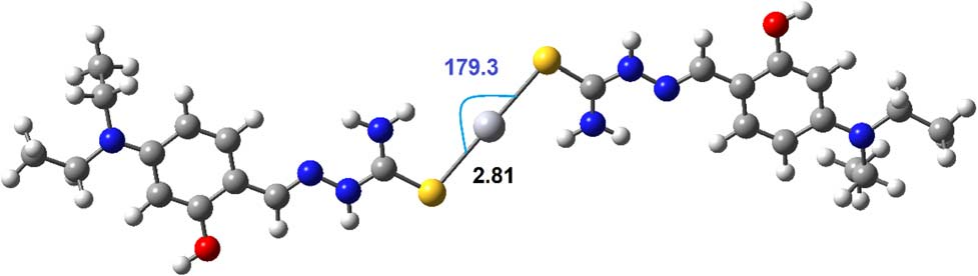
The stable structure of Hg(DST)_2_ sensor at the DFT/PBE0/lanl2dz.

The stable structure of the complex between DST and Hg^2+^ in a 2 : 1 molar ratio was determined at the DFT/PBE0/Lanl2dz and is presented in Fig. 3. The calculation results show that the Hg(DST)_2_ complex is stabilized by two Hg⋯S interactions, with a contact distance of 2.81 Å, which is significantly smaller than the van der Waals radius sum of Hg and S atoms, which is 3.35 Å. The Hg⋯S⋯Hg bond angle is nearly linear, with a value of 179.3°.^51^ The two DST molecules in the complex are not in the same plane but lie in two parallel planes (considering only the π-conjugated system plane).

**Fig. 4:**
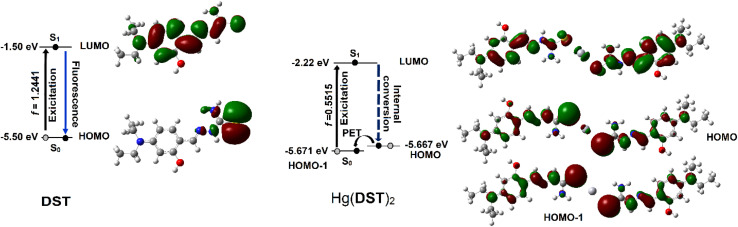
Diagram illustrating the singlet electron transition processes in the excited state for DST and Hg(DST)_2_.

The results of the study on the singlet electron transition process from the ground state S_0_ to the excited state S_1_ (Fig. 4) indicated that in the DST compound, this process was determined by the electron transition from HOMO to LUMO (oscillator strength *f* = 1.2441). Meanwhile, in the Hg(DST)_2_ complex, this process was determined by the electron transition from HOMO − 1 to LUMO (*f* = 0.5515). Therefore, in the excited state, the Hg(DST)_2_ complex underwent a photoinduced electron transfer (PET) process with the transfer of an electron from HOMO to HOMO − 1, leading to the absence of an excited electron transition from LUMO to HOMO − 1 (which would normally result in energy emission in the form of fluorescence).^51–54^ Instead, there was an internal conversion process from LUMO to HOMO, resulting in the quenching of fluorescence in the Hg(DST)_2_ complex.

### 3.2. Application of the Hg(DST)_2_ sensor for detecting biothiols

#### 3.2.1. Determination of the stability constant of the Hg(DST)_2_ complex

To investigate the potential application of the Hg(DST)_2_ sensor for detecting biothiols, the experimental stability constant of this complex was first determined using the nonlinear curve-fitting method, based on data obtained from the fluorescence titration of DST with Hg^2+^.^55^ Here, the complex formation reaction is represented in eqn (1), and the stability constant of the complex (*β*_ex_) is given in eqn (2).

1 Hg^2+^ + 2DST = Hg(DST)_2_

2
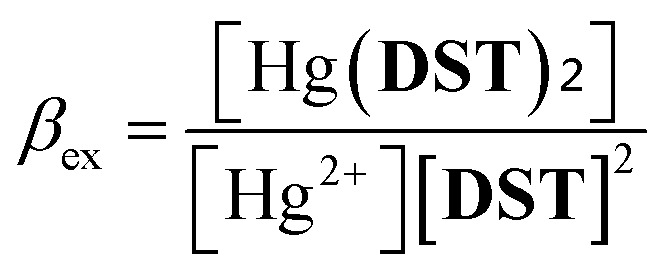


If the initial concentration of DST is denoted as *C*_L_, the initial fluorescence intensity of the DST solution as *F*_0_, and the fluorescence intensity of the DST solution at equilibrium as *F*, then let *x* = *F*/*F*_0_.The concentration of Hg^2+^ used is denoted as *y* = *C*_M_. At equilibrium, the concentrations are expressed as follows:3
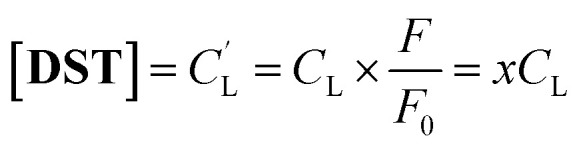
4
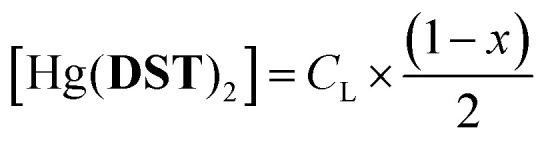
5
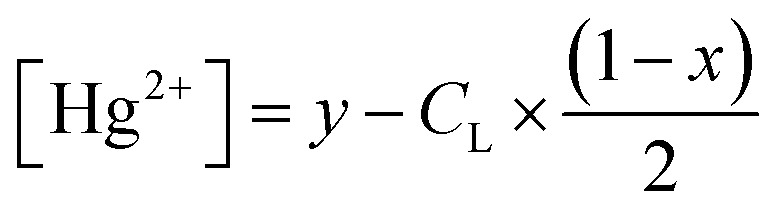


Substituting into eqn (2) yields (6) as follows:6
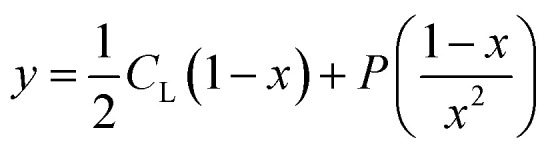
where:7
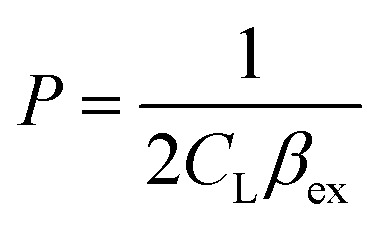
when using *C*_L_ = 20 μM, eqn (6) and (7) become eqn (8) and (9) as follows:8
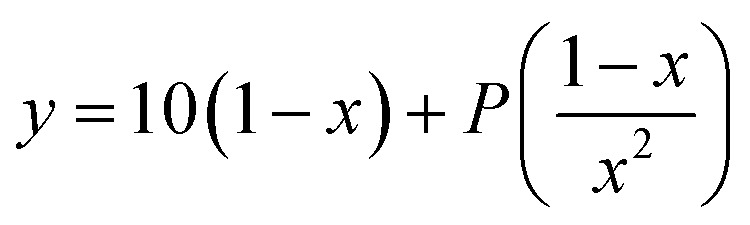
where:9
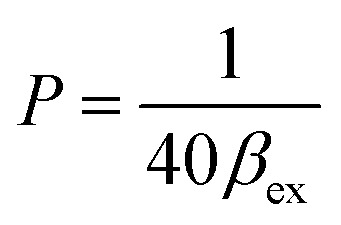


**Fig. 5:**
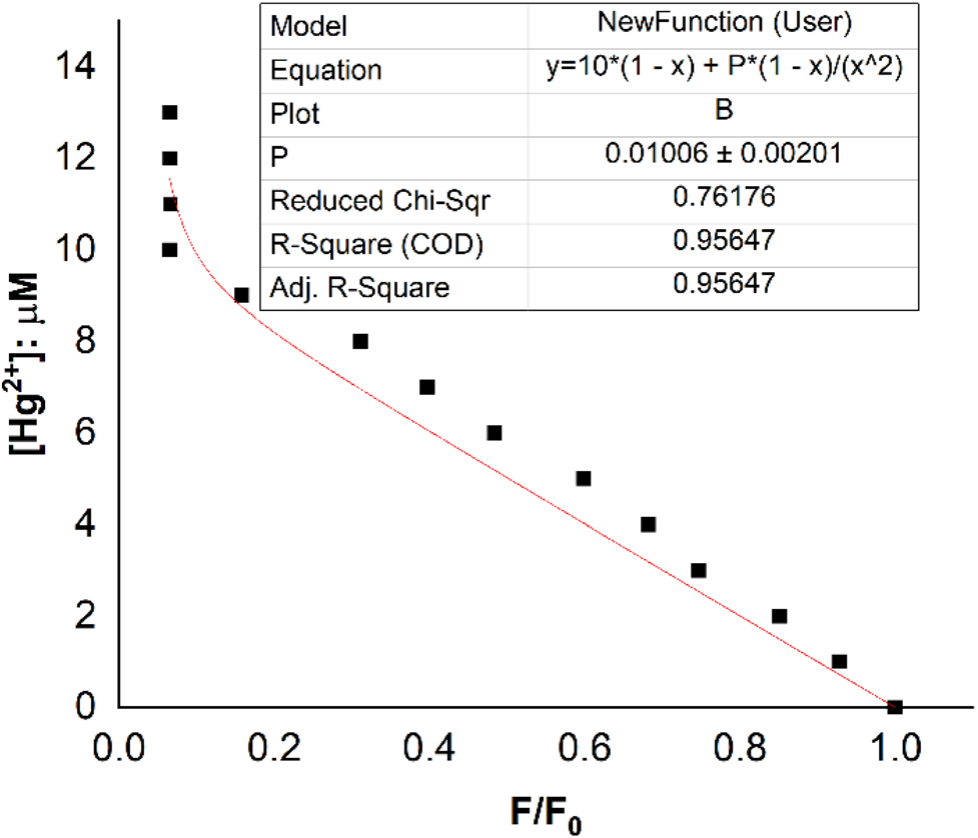
Nonlinear curve fitting for determining the complexation equilibrium constants of the Hg(DST)_2_ complex in an aqueous solution. Where *y* = CM represents the total concentration of Hg^2+^ ions added to the solution, with values of 0, 1, 2, 3, 4, 5, 6, 7, 8, 9, 10, 11, 12, and 13 μM. The initial concentration of DST is *C*_L_ = 20 μM. *F*_0_ and *F* are the fluorescence intensities of the DST solution when the Hg^2+^ concentration is 0 and *C*_M_, respectively. The reaction occurs in a pH 7.2 phosphate buffer, with an excitation wavelength of 368 nm and an emission wavelength of 445 nm.

The fluorescence titration results of the DST solution (*C*_L_ = 20 μM) with Hg^2+^ (concentrations of 0, 1, 2, 3, 4, 5, 6, 7, 8, 9, 10, 11, 12, and 13 μM) and the nonlinear curve fitted to eqn (8) are presented in Fig. 5. The *P* value obtained from this process is 0.01006 ± 0.00201. From eqn (9), the calculated stability constant *β*_ex_ is 2.458 μM^−1^ (or 10^0.395^ μM^−1^, equivalent to 10^12.395^ M^−1^).

### 3.3. Application of the Hg(DST)_2_ sensor for the detection of biothiols

The potential application of the Hg(DST)_2_ complex as a fluorescence sensor for detecting GSH, Cys, and Hcy is based on the complex exchange reaction, which depends on the stability constants of the complexes between Hg^2+^ and the ligands GSH, Cys, Hcy, and DST. For GSH (written as the ligand H_3_L), the complexes with Hg^2+^ are detected as ML, MLH, MLH_2_, ML_2_, ML_2_H, ML_2_H_2_, and ML_2_H_3_, with corresponding log *β*_ex_ values of 26.04, 32.49, 35.68, 41.58, 42.40, 52.29, and 55.28, respectively.^56,57^ For Cys (written as the ligand H_2_L′), the complexes with Hg^2+^ are detected as 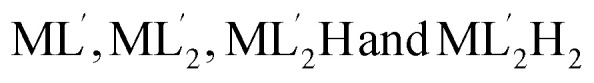, with corresponding log *β*_ex_ values of 14.21, 43.57, 54.37, and 61.79, respectively.^56,58–60^ For Hcy (written as the ligand H_2_L′′), there are still very few published studies on the composition and stability constants of its complexes with Hg^2+^. One study has identified the existence of the 
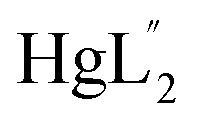
 complex, with a stability constant log *β*_ex_ determined to be 39.4.^61^ In general, previous studies have shown that Hg^2+^ complexes with GSH, Cys, and Hcy exist in various forms, depending on the environment and their concentration ratios. At neutral pH, the predominant species are 
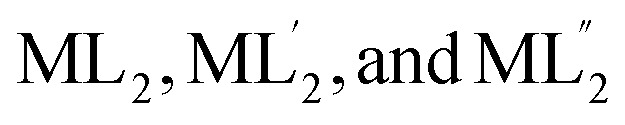
. Specifically, for GSH, even at a GSH : Hg^2+^ ratio of up to 22 : 1, the ML_2_ form still accounts for 95%. For Cys, the proportions of other complexes increase, but 
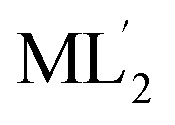
 remains the dominant species.^61^

According to the calculations above, the log *β*_ex_of the Hg(DST)_2_ complex is 12.395, much smaller than the log *β*_ex_ of the 
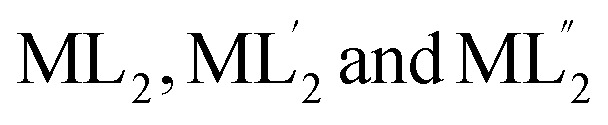
 complexes, which are 41.58, 43.57, and 39.4, respectively. Therefore, theoretically, GSH, Cys, and Hcy can react with Hg(DST)_2_ to form corresponding complexes and release free DST, altering the fluorescence intensity of the solution. In other words, Hg(DST)_2_ can be used as a fluorescence sensor to detect GSH, Cys, and Hcy. One noteworthy point is that since the log *β*_ex_ values of the 
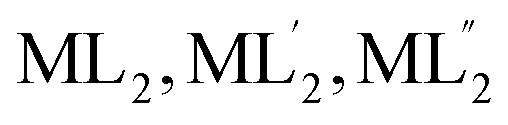
 complexes are approximately similar, it is unlikely that Hg(DST)_2_ can selectively detect each thiol in the GSH, Cys, and Hcy group.

**Fig. 6:**
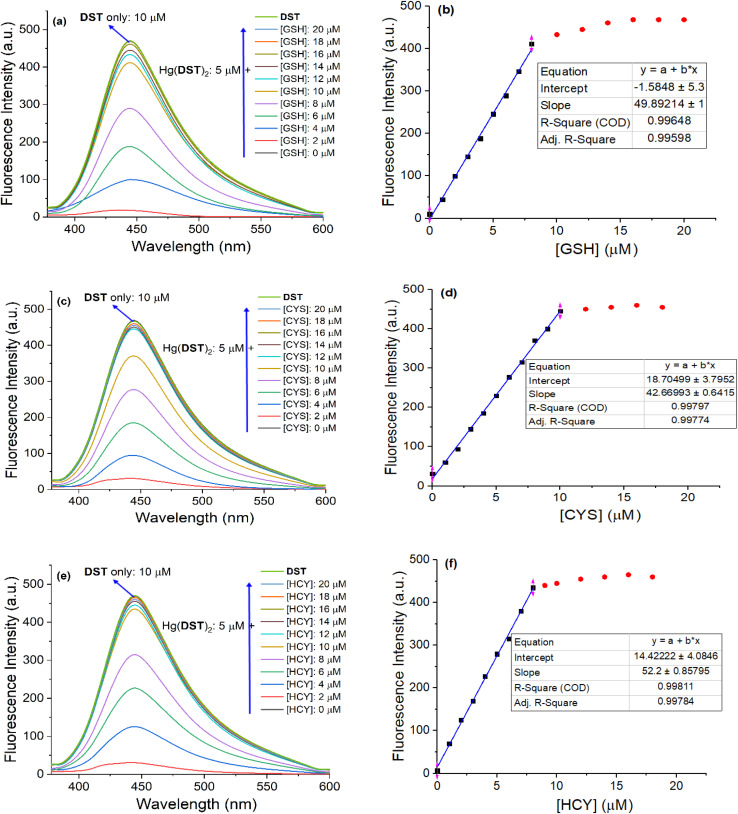
Fluorescence titration results of the Hg(DST)_2_ complex sensor solution (5 μM) by the thiols GSH, Cys, and Hcy (0, 1, 2, 3, 4, 5, 6, 7, 8, 9, 10, 12, 14, 16, 18, 20 μM) in a pH 7.2 phosphate buffer, with an excitation wavelength of 368 nm and an emission wavelength of 445 nm. GSH (a and b), Cys (c and d), and Hcy (e and f).

Indeed, the experimental results on the fluorescence titration of the Hg(DST)_2_ sensor solution by the thiols GSH, Cys, and Hcy, as presented in Fig. 6, are entirely consistent with the aforementioned observations. All three thiols – GSH, Cys, and Hcy – react with Hg(DST)_2_, releasing DST and leading to an increase in the fluorescence intensity of the solution (The images showing the fluorescence color changes of the Hg(DST)_2_ solution in the presence of biothiols, taken inside the fluorescence spectrophotometer chamber, are presented in Fig. S5†). When the concentration of the thiols approaches approximately twice the concentration of Hg(DST)_2_ used, as well as beyond this point, the fluorescence intensity of the solution remains nearly unchanged. This indicates that the complexes formed between Hg^2+^ and GSH, Cys, and Hcy are predominantly in the 
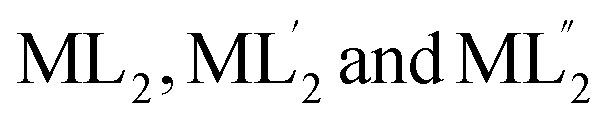
 forms, as previously noted.

The survey results indicate that the Hg(DST)_2_ complex sensor can quantitatively detect GSH, Cys, and Hcy, as evidenced by the excellent linear relationship between the fluorescence intensity of the solution and the concentrations of GSH, Cys, and Hcy within a certain range. Accordingly, the linear ranges for determining GSH, Cys, and Hcy using the Hg(DST)_2_ sensor are 0.34–8.00 μM, 0.47–10.00 μM, and 0.26–8.0 μM, respectively, with corresponding linear correlation coefficients (*R*) of approximately 0.998. Among these, the values of 0.34, 0.47, and 0.26 μM represent the detection limits of GSH, Cys, and Hcy, respectively, using the Hg(DST)_2_ sensor, as determined from the calibration curve equation at low concentrations (see Fig. S6 and Table. S1 of the ESI†).^62^ Compared to some fluorescent sensors for thiol detection based on metal ion complexes reported in recent times (Table 1), the Hg(DST)_2_ sensor had a comparable LOD for thiols but had the advantage of operating in a fully aqueous environment.

**Fig. 7:**
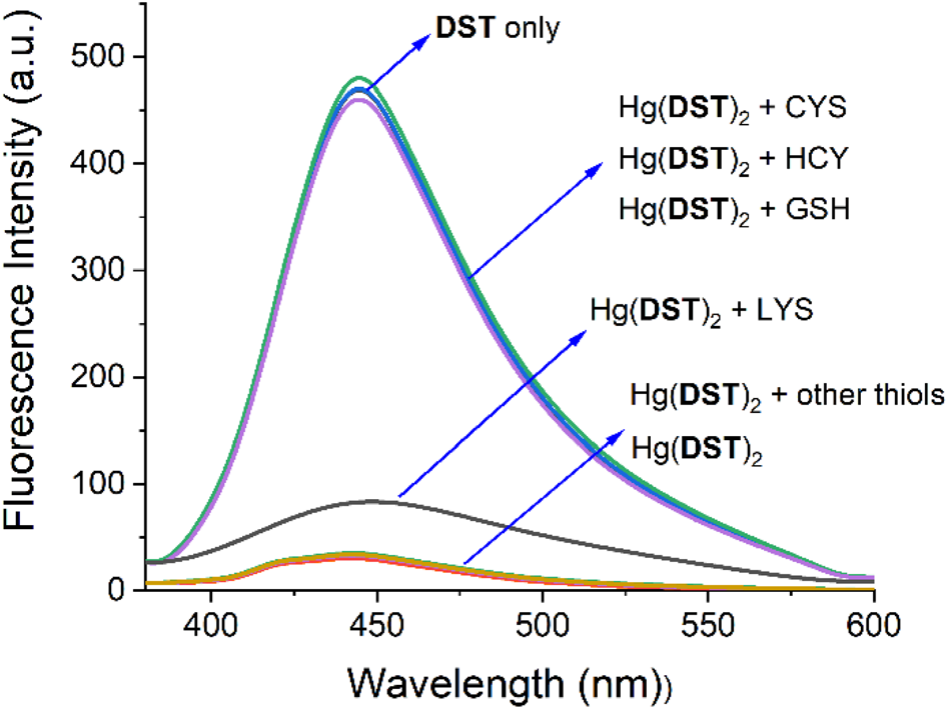
Fluorescence spectra of the Hg(DST)_2_ solution (5 μM) upon addition of thiols GSH, Cys, and Hcy (20 μM each), as well as amino acids Ala, Arg, Asp, Glu, Gly, His, Ile, Leu, Lys, Met, Ser, Thr, Trp, Tyr and Val (20 μM each), in a pH 7.2 phosphate buffer, with an excitation wavelength of 368 nm and an emission wavelength of 445 nm.

The influence of amino acids on the use of the Hg(DST)_2_ sensor for thiol detection was also investigated and presented in Fig. 7. The results showed that, except for Lys (lysine), which altered the fluorescence spectrum of the Hg(DST)_2_ sensor solution, the other amino acids, including Ala (alanine), Arg (arginine), Asp (aspartic acid), Glu (glutamic acid), Gly (glycine), His (histidine), Ile (isoleucine), Leu (leucine), Met (methionine), Ser (serine), Thr (threonine), Trp (tryptophan), Tyr (tyrosine), and Val (valine), had little to no effect on the fluorescence spectrum of the Hg(DST)_2_ sensor solution. These results indicated that the Hg(DST)_2_ sensor could detect the thiols GSH, Cys, and Hcy in the presence of the above amino acids, except for Lys. Additionally, the investigation results showed that the Hg(DST)_2_ sensor was unable to detect individual thiols within the GSH, Cys, and Hcy groups separately. This finding was consistent with previous reports on fluorescent sensors for thiol detection based on metal ion complexes. However, this limitation did not diminish the applicability of such sensors, as thiols typically do not coexist in equal concentrations in real samples. For instance, in human whole blood samples, GSH is significantly higher than other thiols (sometimes up to 1 mM), whereas, in human plasma samples, Cys is much more abundant than other thiols (sometimes reaching up to 250 μM).^63–65^

**Fig. 8:**
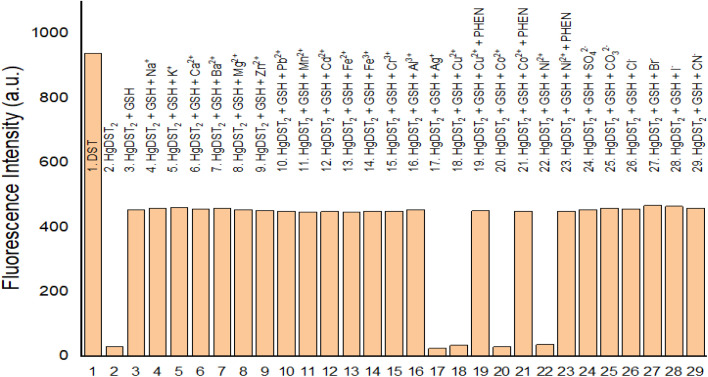
Fluorescence intensity of solutions: DST (20 μM); Hg(DST)_2_ (10 μM); Hg(DST)_2_ (10 μM) + GSH (10 μM); Hg(DST)_2_ (10 μM) + GSH (10 μM) + metal ions or anions (20 μM); Hg(DST)_2_ (10 μM) + GSH (10 μM) + Cu^2+^, Co^2+^or Ni^2+^ (20 μM) + PHEN (1500 μM); in a pH 7.2 phosphate buffer, with an excitation wavelength of 368 nm and an emission wavelength of 445 nm.

The use of the Hg(DST)_2_ complex fluorescent sensor for detecting the thiols GSH, Cys, and Hcy was also investigated for its susceptibility to interference by various ions, including alkali metal ions (Na^+^, K^+^), alkaline earth metal ions (Ca^2+^, Ba^2+^, Mg^2+^), transition metal ions (Ag^+^, Cu^2+^, Co^2+^, Ni^2+^, Mn^2+^, Fe^2+^, Fe^3+^, Cr^3+^), other metal ions (Zn^2+^, Pb^2+^, Cd^2+^, Al^3+^), as well as common anions such as sulfate (SO_4_^2−^), carbonate (CO_3_^2−^), chloride (Cl^−^), bromide (Br^−^), iodide (I^−^), and cyanide (CN^−^). The results, presented in Fig. 8, S7, and S8† (in the ESI†), demonstrated that, except for Ag^+^, Cu^2+^, Co^2+^, and Ni^2+^, the presence of the other ions did not affect the thiol detection method using the Hg(DST)_2_ sensor, as evidenced by the absence of significant fluorescence intensity changes in (Hg(DST)_2_ + thiol) solutions. The interference caused by Cu^2+^, Co^2+^, and Ni^2+^ could be eliminated by the complexing agent 1,10-phenanthroline (PHEN). Meanwhile, a suitable complexing agent to mitigate the interference of Ag^+^ has not yet been identified. However, since Ag^+^ is not a biological metal, its interference in thiol detection in biological samples is not a major concern.

**Scheme 1:**
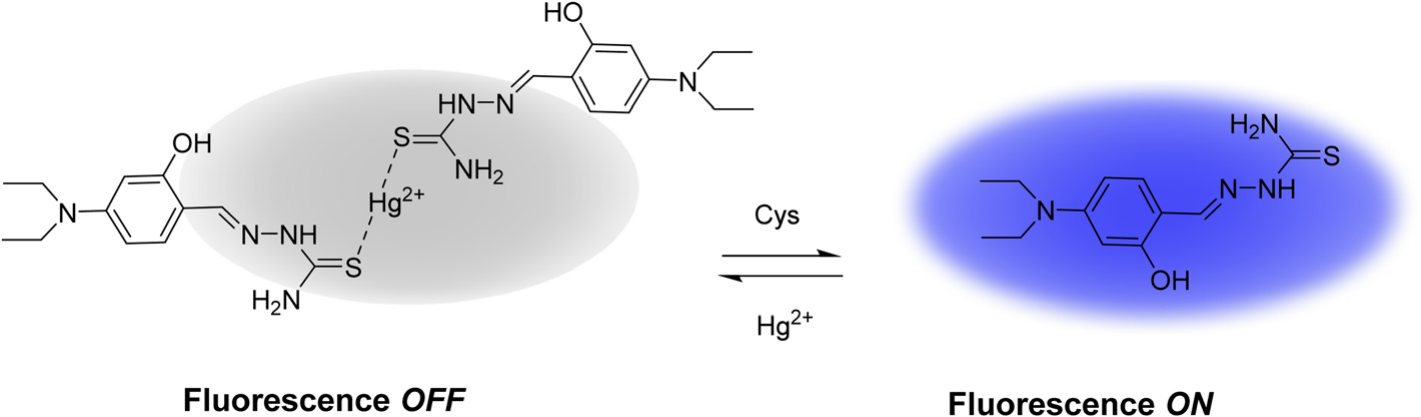
Diagram Illustrating the operating principle of the Hg(DST)_2_ sensor with biothiols.

The reaction time between the Hg(DST)_2_ complex and the biothiols GSH, Cys, and Hcy was almost instantaneous. After 1 minute, the fluorescence intensity of the solution had nearly stabilized. The sensing mechanism is summarized in Scheme 1.

## 4. Conclusions

This study presents the successful development and validation of the Hg(DST)_2_ fluorescent sensor for the detection of thiols (GSH, Cys, and Hcy) in aqueous media, achieving detection limits of 0.34, 0.47, and 0.26 μM, respectively. The sensor exhibited robust selectivity against most common amino acids, metal ions, and anions, with interference from Cu^2+^, Co^2+^, and Ni^2+^ effectively neutralized using 1,10-phenanthroline (PHEN), while Ag^+^ interference remains negligible in biological contexts due to its non-physiological relevance. Compared to existing metal-complex-based fluorescent sensors, Hg(DST)_2_ offers comparable sensitivity and the distinct advantage of operating in a fully aqueous environment. These attributes position Hg(DST)_2_ as a highly effective and practical tool for real-time thiol monitoring in biomedical diagnostics (*e.g.*, oxidative stress assessment) and environmental analysis.

## Conflicts of interest

There are no conflicts to declare.

## Supplementary Material

RA-015-D5RA02268A-s001

## Data Availability

The data supporting this article have been included as part of the ESI.†
