# Supplementary material for: A mercury complex-based fluorescent sensor for biological thiols
Source: RSC Adv. 2025 Jun 13;15(25):20125–33. doi: 10.1039/d5ra02268a (PMC12163708; doi:10.1039/d5ra02268a)
Supplement: RA-015-D5RA02268A-s001 [file RA-015-D5RA02268A-s001.pdf]

Supplementary Information (SI) for RSC Advances.
This journal is © The Royal Society of Chemistry 2025

Supplementary Data for

A Mercury Complex-Based Fluorescent Sensor for Biological Thiols

Nguyen Khoa Hien[1], Trinh Thi Giao Chau[1], Nguyen Dinh Luyen[2], Quan V. Vo[3], Mai Van Bay[4], Son Tung Ngo[5,6], Pham Cam Nam[7*], and Duong Tuan Quang[2*]

[1]Mientrung Institute for Scientific Research, Vietnam National Museum of Nature, Vietnam Academy of Science and Technology, Hue 49000, Vietnam

[2]Department of Chemistry, Hue University, Hue 49000, Vietnam

[3]Faculty of Chemical Technology-Environment, The University of Danang - University of Technology and Education, 48 Cao Thang, Danang 50000, Vietnam

[4]The University of Danang, University of Science and Education, Danang 50000, Vietnam

[5]Laboratory of Biophysics, Institute for Advanced Study in Technology, Ton Duc Thang University, Ho Chi Minh City 72915, Vietnam

[6]Faculty of Pharmacy, Ton Duc Thang University, Ho Chi Minh City 72915, Vietnam

[7]The University of Danang – University of Science and Technology, Danang 50000, Vietnam

*Corresponding author: duongtuanquang@hueuni.edu.vn; pcnam@dut.udn.vn

1. Structural characteristics of the fluorescent compound DST

1.1. ^{1}H NMR spectra of the fluorescent compound DST

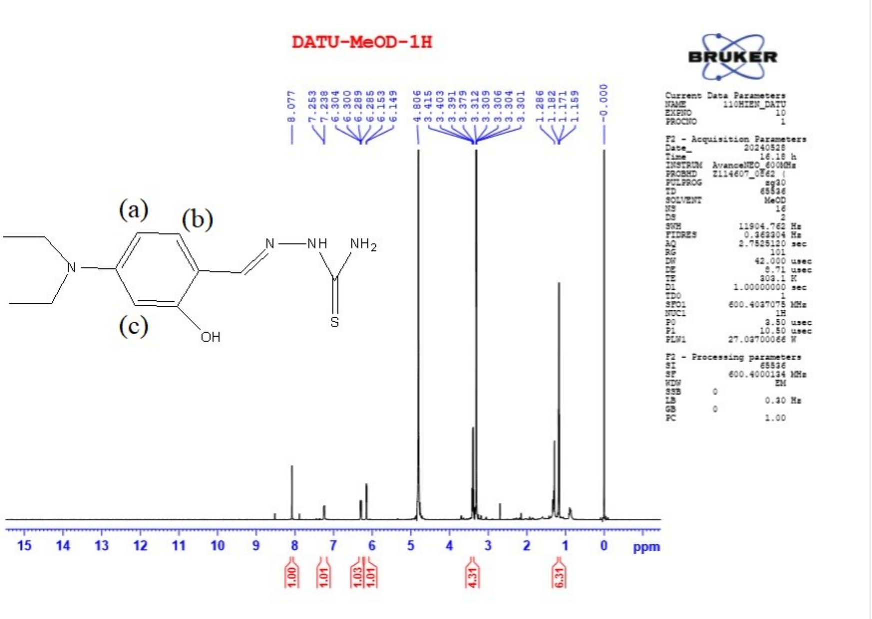

Fig.S1(a). *^{1}H NMR spectra of the fluorescent compound* ***DST***

^{1}H NMR (600 MHz, MeOD): C**H**$_3$-CH$_2$-(6H, triplet, 1.171 ppm, J=7.2 Hz), CH$_3$-C**H**$_2$-(4H, quartet, 3.397 ppm, J=7.2 Hz), aromatic **H**(c) (1H, doublet, 6.151 ppm, J=2.4 Hz), aromatic

H (a) (1H, doublet of doublet, 6.295 ppm, J=2.4 Hz, J=9.0 Hz), aromatic **H** (b) (1H, 7.246 ppm, J=9.0 Hz), -C**H**=N- (1H, singlet, 8.077 ppm) (Fig.S1).

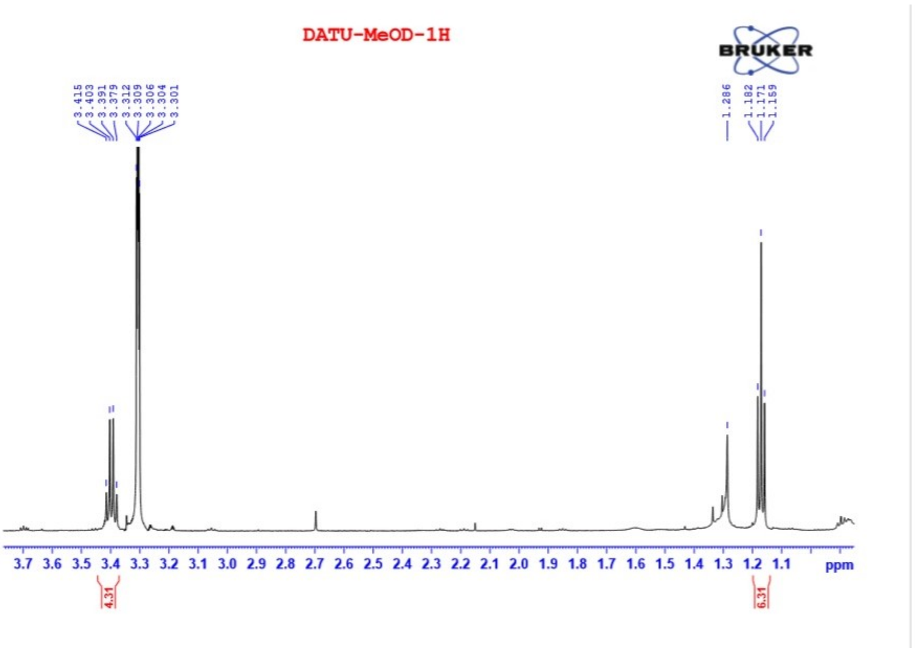

Fig.S1(b). *^{1}H NMR spectra of the fluorescent compound* ***DST*** *(continued)*

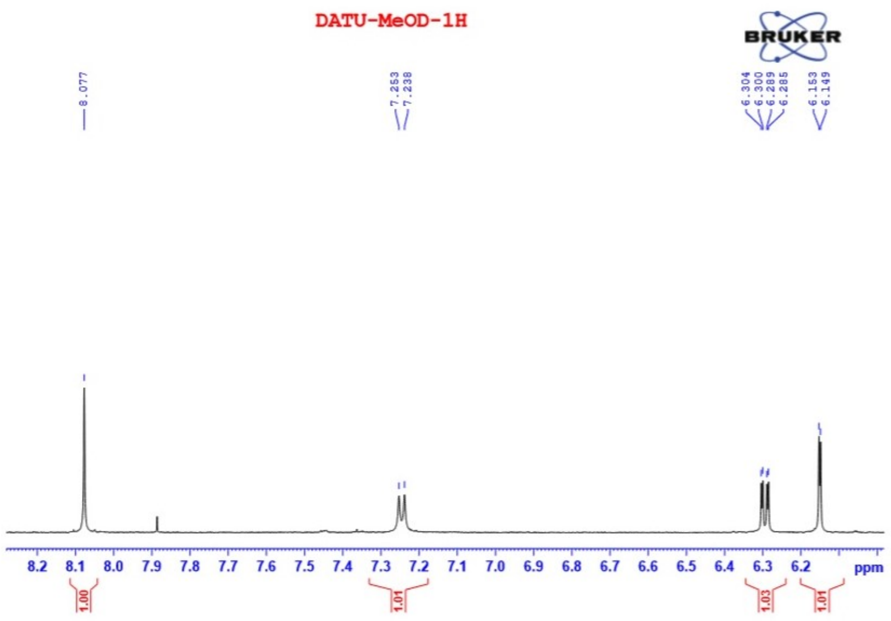

Fig.S1(c). *^{1}H NMR spectra of the fluorescent compound* ***DST*** *(continued)*

1.2. ^{13}C NMR spectra of the fluorescent compound DST

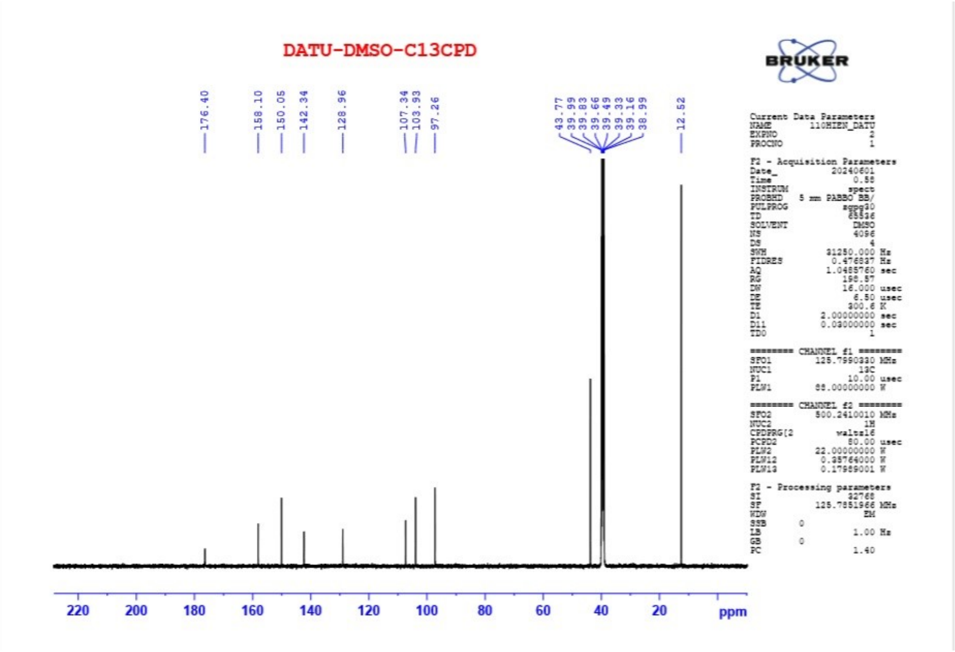

Fig.S2. *^{13}C NMR spectra of the fluorescent compound* ***DST***

^{13}C NMR (150 MHz, DMSO): N-**C**=S (1C; 176.40 ppm), **C**-OH (1C; 158.10 ppm), $(C_2H_5)_2$N-**C** (1C, 150.05 ppm), **C**=N (1C, 142.34), other aromatic **C** (4C, 128.96, 107.34, 103.93, and 97.26 ppm), -**C**H_2-N (2C, 43.77 ppm), **C**H_3- (2C, 12.52 ppm) (Fig.S2).

1.3. MS spectra of the fluorescent compound DST

MS (M+H$^+$): m/z=267.1 (Fig.S3). Calcd for $C_{12}H_{18}N_4OS$ (M=266.1)

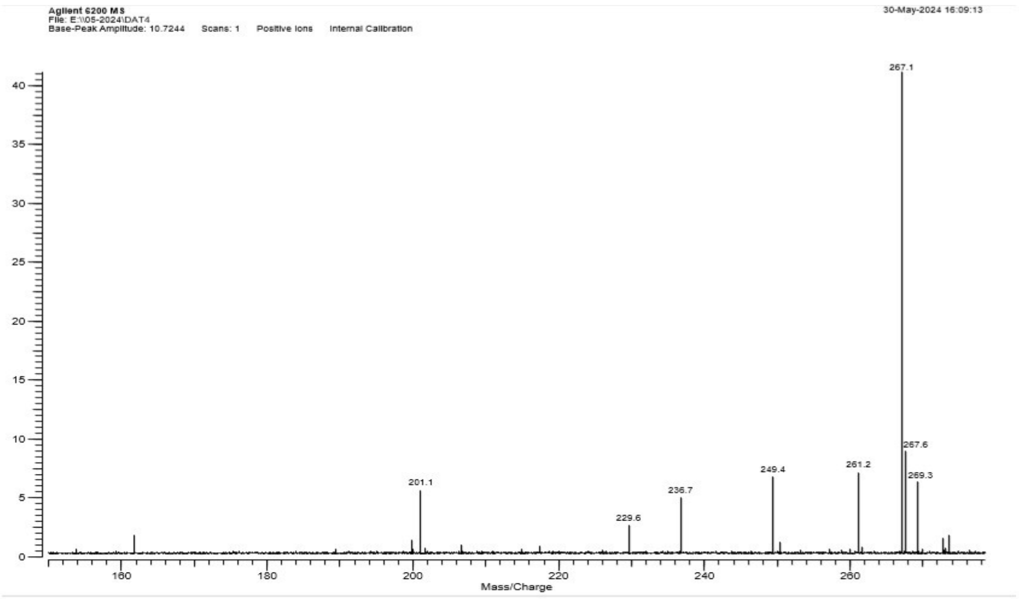

Fig.S3. *MS spectra of the fluorescent compound* ***DST***

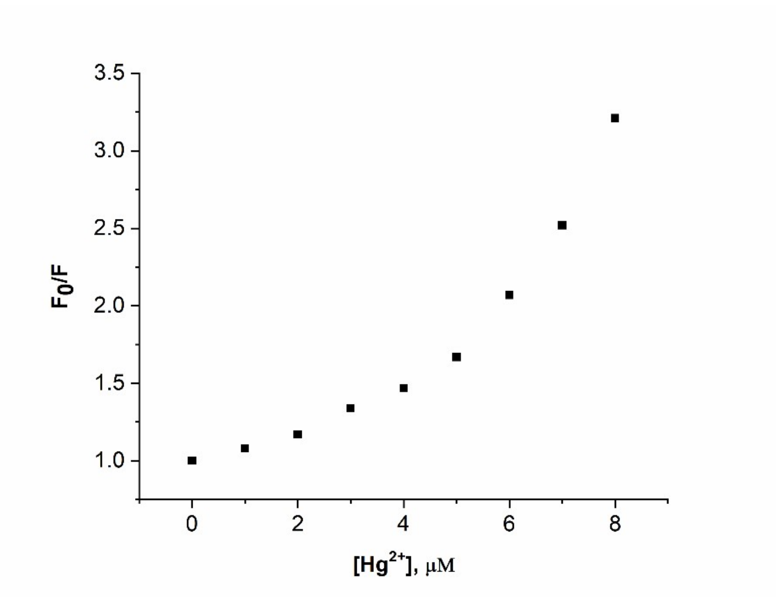

Fig.S4. *The Stern–Volmer plot for quenching process of **DST** by Hg^{2+} (**DST** 20 µM, Hg^{2+} at concentrations ranging from 0 to 8.0 µM, in pH 7.2 phosphate buffer, with an excitation wavelength of 368 nm, an emission wavelength of 445 nm)*

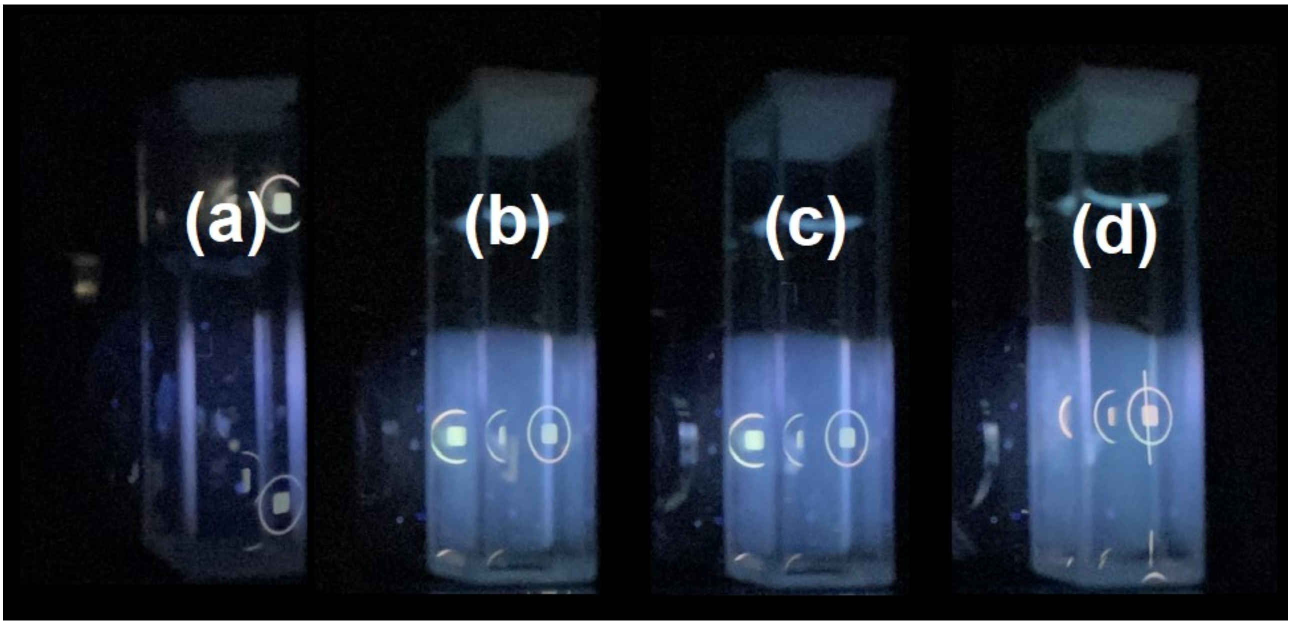

Fig.S5. *The images showing the fluorescence color changes of the Hg(**DST**)$_2$ solution in the presence of biothiols, taken inside the fluorescence spectrophotometer chamber: (a) Hg(**DST**)$_2$ 5 µM, (b) Hg(**DST**)$_2$ 5 µM + GSH 20µM, (c) Hg(**DST**)$_2$ 5 µM + Cys 20µM, (d) Hg(**DST**)$_2$ 5 µM + Hcy 20µM*

2. Application of the $Hg(DST)_2$ sensor for the detection of biothiols

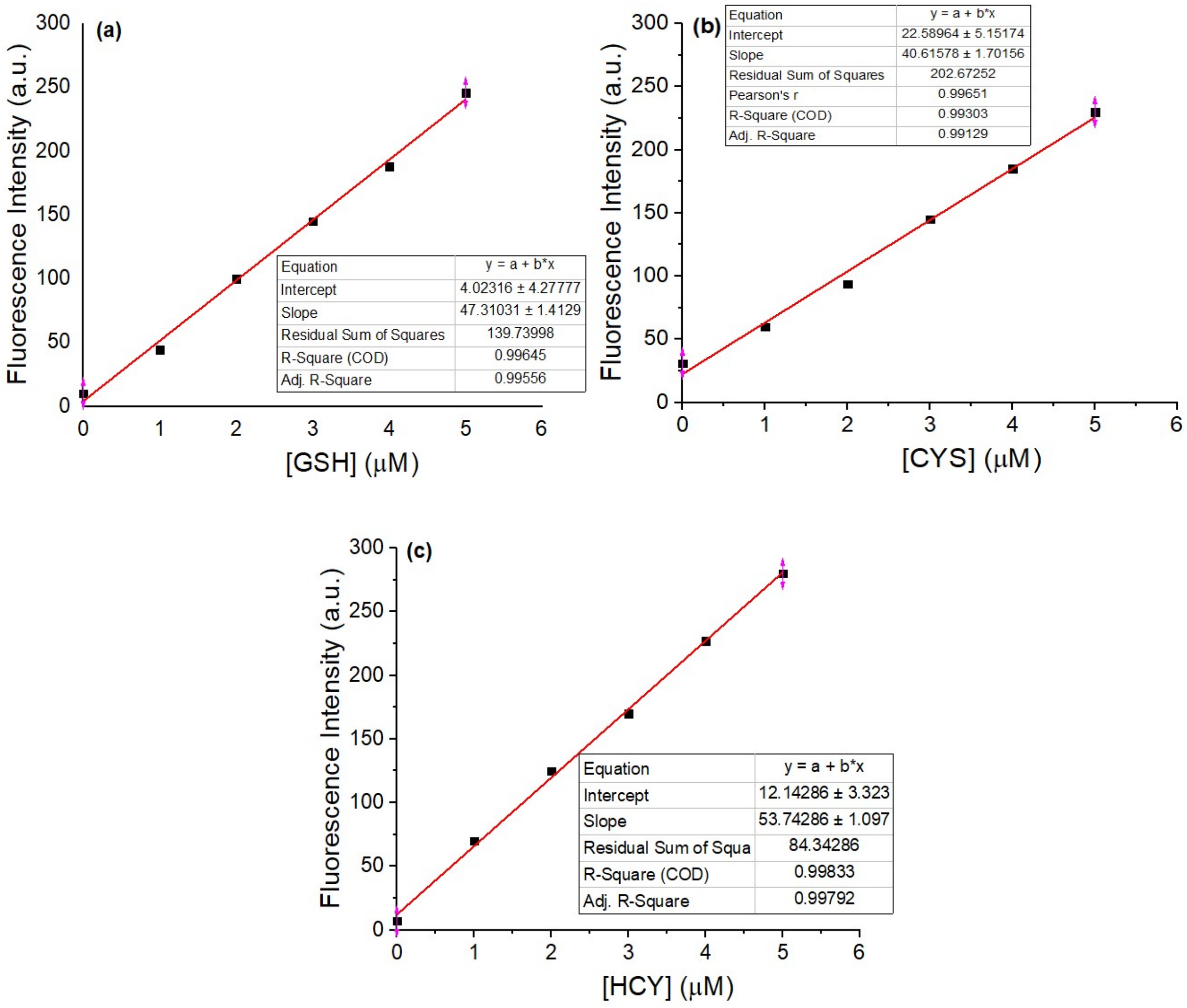

Fig.S6. *The calibration curve determining LOD: Hg(**DST)₂** (5μM) in aqueous solution (pH 7.2 phosphate buffer) vs the concentration of GSH (0-5 μM), Cys (0-5 μM), and Hcy (0-5 μM), at an excitation wavelength of 368 nm and an emission wavelength of 445 nm*

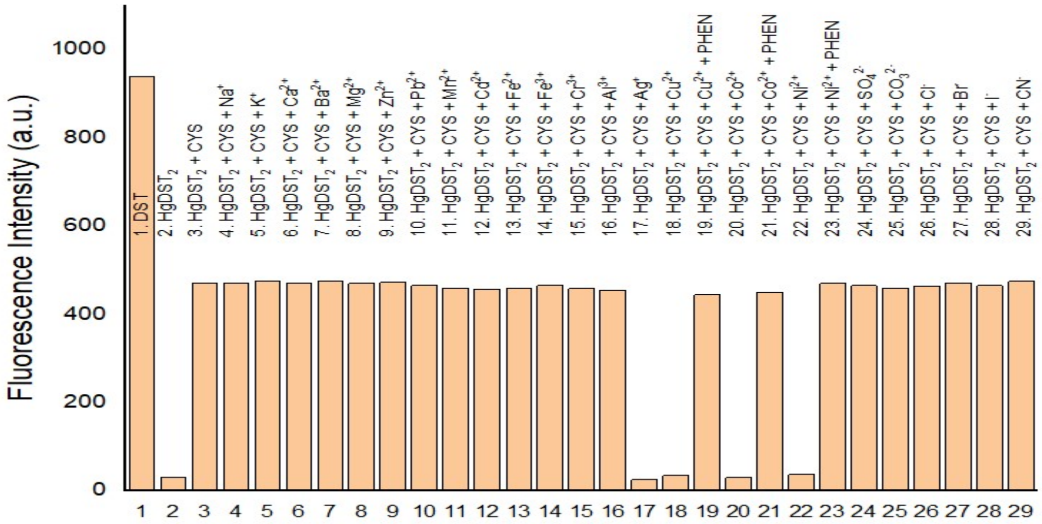

Fig.S7. *Fluorescence intensity of solutions: **DST**(20 μM); Hg(**DST)**$_2$ (10 μM); Hg(**DST)**$_2$ (10 μM)+GSH(10 μM); Hg(**DST)**$_2$ (10 μM) +CYS(10 μM) + metal ions or anions (20 μM); Hg(**DST)**$_2$ (10 μM)+GSH(10 μM) + Cu^{2+}, Co^{2+}or Ni^{2+} (20 μM) + PHEN (1500 μM); in a pH 7.2 phosphate buffer, with an excitation wavelength of 368 nm and an emission wavelength of 445 nm.*

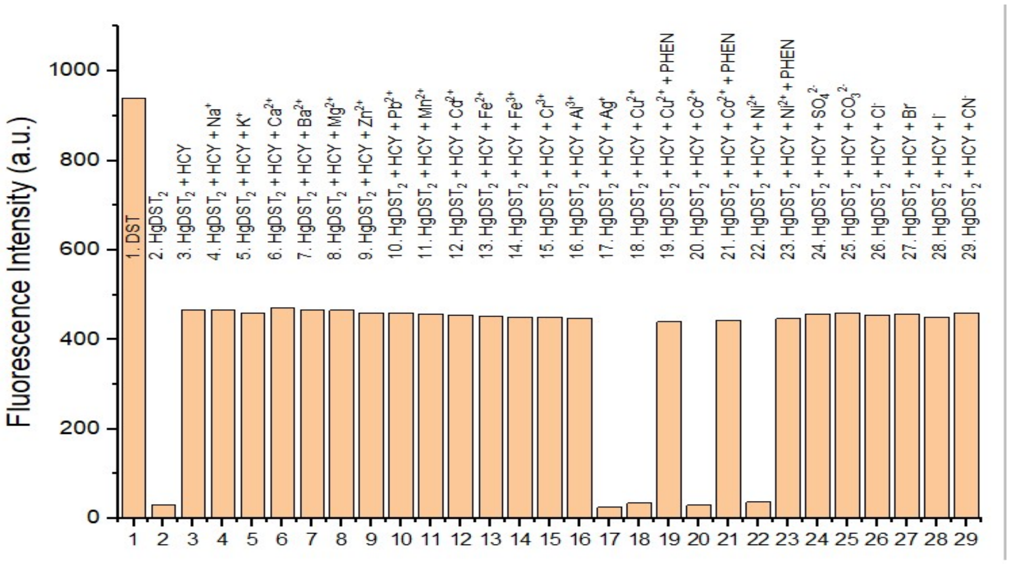

Fig.S8. *Fluorescence intensity of solutions: **DST**(20 μM); Hg(**DST**)$_2$ (10 μM); Hg(**DST**)$_2$(10 μM) + GSH(10 μM); Hg(**DST**)$_2$(10 μM) + HCY(10 μM) + metal ions or anions(20 μM); Hg(**DST**)$_2$ (10 μM) + GSH(10 μM) + Cu^{2+}, Co^{2+}or Ni^{2+} (20 μM) + PHEN(1500 μM); in a pH 7.2 phosphate buffer, with an excitation wavelength of 368 nm and an emission wavelength of 445 nm.*

Table.S1. *Details of calculation of LOD of methods for detecting the thiols GSH, Cys, and Hcy using the Hg(**DST**)$_2$ sensor*

**Thiol**	RSS	SD	B	LOD=3*SD/B (μM)
**GSH**	139.74	5.29	47.31	0.34
**CYS**	202.67	6.37	40.62	0.47
**HCY**	84.34	4.59	53.74	0.26
